# Comparative Systems Analyses Reveal Molecular Signatures of Clinically tested Vaccine Adjuvants

**DOI:** 10.1038/srep39097

**Published:** 2016-12-13

**Authors:** Thorunn A. Olafsdottir, Madelene Lindqvist, Intawat Nookaew, Peter Andersen, Jeroen Maertzdorf, Josefine Persson, Dennis Christensen, Yuan Zhang, Jenna Anderson, Sakda Khoomrung, Partho Sen, Else Marie Agger, Rhea Coler, Darrick Carter, Andreas Meinke, Rino Rappuoli, Stefan H. E. Kaufmann, Steven G. Reed, Ali M. Harandi

**Affiliations:** 1 Department of Microbiology and Immunology, Institute of Biomedicine, Sahlgrenska Academy, University of Gothenburg, Gothenburg, Sweden; 2 Department of Biology and Biological Engineering, Chalmers, University of Technology, Gothenburg, Sweden; 3 Department of Biomedical Informatics, College of Medicine, University of Arkansas for Medical Sciences, Little Rock, Arkansas, USA; 4 Department of Infectious Disease Immunology, Statens Serum Institut, Copenhagen, Denmark; 5 Department of Immunology, Max Planck Institute for Infection Biology, Berlin, Germany; 6 Infectious Disease Research Institute, Seattle, Washington, USA; 7 Valneva Austria GmbH, Campus Vienna Biocenter, Vienna, Austria; 8 GSK Vaccines, Siena, Italy

## Abstract

A better understanding of the mechanisms of action of human adjuvants could inform a rational development of next generation vaccines for human use. Here, we exploited a genome wide transcriptomics analysis combined with a systems biology approach to determine the molecular signatures induced by four clinically tested vaccine adjuvants, namely CAF01, IC31, GLA-SE and Alum in mice. We report signature molecules, pathways, gene modules and networks, which are shared by or otherwise exclusive to these clinical-grade adjuvants in whole blood and draining lymph nodes of mice. Intriguingly, co-expression analysis revealed blood gene modules highly enriched for molecules with documented roles in T follicular helper (TFH) and germinal center (GC) responses. We could show that all adjuvants enhanced, although with different magnitude and kinetics, TFH and GC B cell responses in draining lymph nodes. These results represent, to our knowledge, the first comparative systems analysis of clinically tested vaccine adjuvants that may provide new insights into the mechanisms of action of human adjuvants.

Most vaccine antigens currently under investigation are based on highly purified subunits of pathogens or recombinant proteins and thus possess improved safety profiles. However, the shift from whole live attenuated or killed pathogens toward subunit vaccines necessitates the use of vaccine adjuvants that enhance, accelerate, prolong and/or modulate the desired immune responses^1^,^2^ for different pathogens and vaccinees^3^,^4^. The rational development of new vaccines thus requires a thorough understanding of the modes of action of human vaccines and adjuvants.

Recently, systems biology approaches have successfully been used to study the mechanisms of action of a few vaccines approved for use in humans^5^ and, more recently, to study the modes of action of vaccine adjuvants (reviewed in ref. 6). However, the different experimental conditions used in adjuvant research often make it difficult to make direct comparisons. Furthermore, the most promising vaccine adjuvants can be difficult to gain access to for comparative analysis due to proprietary rights and conflict of interest issues. Within the EU-funded large collaborative project Advanced Immunization Technologies (ADITEC; grant agreement no.: 280873), an unprecedented opportunity emerged, which enabled a head-to-head comparison of some of the most promising vaccine adjuvants in clinical development. We have recently reported the results from the first comparative immunogenicity and protective efficacy testing of several human adjuvants^7^.

In the present study, we explored the molecular signatures of a panel of three adjuvants in clinical development (CAF01, IC31 and GLA-SE) along with 2% Alhydrogel (Alum), the most commonly used adjuvant in licensed vaccines. CAF01 is a two-component liposomal adjuvant formulation composed of cationic liposomes formed of the dimethyldioctadecylammonium surfactant stabilized with trehalose dibehenate, a synthetic analog to mycobacterial cord factor (trehalose dimycolate), and acting through mincle receptor activation^8^,^9^. IC31 is another two-component adjuvant consisting of the cationic peptide KLK combined with the Toll-like receptor 9 (TLR9)-stimulatory oligodeoxynucleotide ODN1a^10^. Glucopyranosyl lipid adjuvant-stable emulsion (GLA-SE) is a synthetic TLR4 agonist in a squalene-based oil-in-water emulsion^11^. Aluminum-based mineral salts have been considered the adjuvant of choice for vaccination against infections that can be prevented by an antibody (Ab) response and have been successfully used in several licensed vaccines^12^,^13^.

As a model antigen for the present study we chose H56, a clinical-grade multistage tuberculosis (TB) vaccine candidate under clinical evaluation (NCT01865487, NCT02375698, NCT02375698, NCT02503839, NCT01967134, NCT02378207). H56 is a fusion protein consisting of two well-characterized early secreted antigens, antigen 85B (Ag85B) and early secreted antigen 6 (ESAT-6) and one latency-associated antigen Rv2660c from *Mycobacterium tuberculosis*^14^. This vaccine candidate has been shown to provide protective immunity in mice, in combination with the novel adjuvants tested in the present study (CAF01, IC31 and GLA-SE)^7^. It has furthermore provided protective immunity against TB in cynomolgus macaques in combination with IC31^15^, and CAF01^16^. H56 is in clinical development in combination with IC31^17^.

Using a genome-wide transcriptomics analysis of whole blood (WB) and draining lymph nodes (dLNs) at early time points after immunization combined with a systems biology approach, we identified both common and unique transcriptional profiles of the four adjuvants tested. Transcriptional changes associated with the induction of adaptive immune responses were commonly detected in WB for all adjuvants, whereas co-expression analysis identified distinct gene modules containing adjuvant-unique molecules that have previously been shown to be involved in germinal center (GC) reactions and/or the skewing of T helper cell responses. Guided by the co-expression analysis data, we therefore studied the ability of the four adjuvants to induce GC formation in the draining LNs following immunization in mice. These data showed that all of these adjuvants, when given in combination with H56 antigen, enhanced TFH and GC B cell responses compared with those of the H56 antigen alone, although kinetics and cell frequency differed among the different adjuvanted groups.

The results reported herein are, to our knowledge, the first comparative systems biology analysis of clinically tested vaccine adjuvants and thus could inform the rational development of vaccine adjuvants for human use.

## Results

### Transcriptomic profiles in WB and dLNs of mice induced by different clinically tested adjuvants

High-quality RNA samples extracted from WB and the dLNs of mice at four different time points (6 h, 24 h, 72 h and 168 h) after a subcutaneous (s.c.) injection of clinical-grade H56 with or without each clinical-grade adjuvant were subjected to genome-wide microarray analysis.

**Figure 1:**
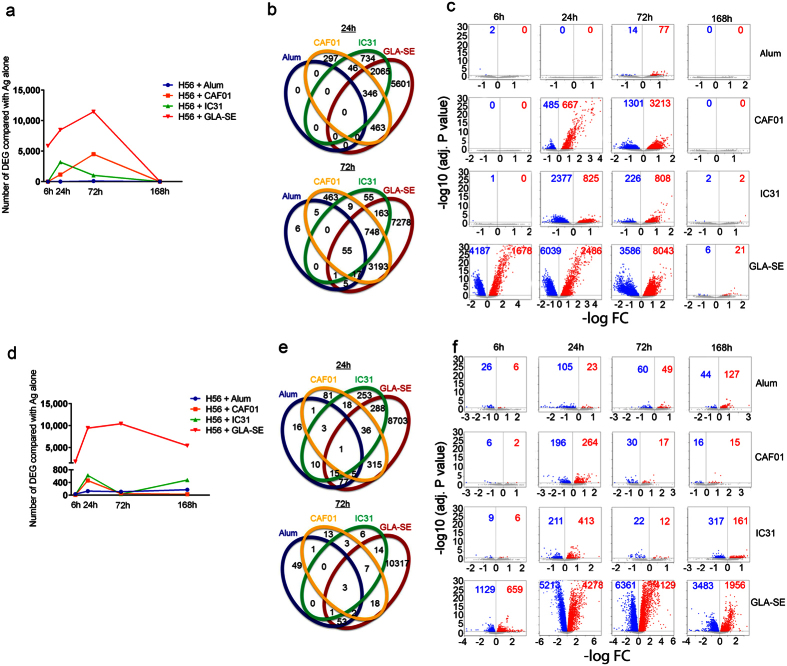
Overview of transcriptomic changes in whole blood and lymph nodes of mice in response to vaccine adjuvants. Groups of mice were immunized with H56 alone or in combination with Alum, CAF01, IC31 or GLA-SE. The numbers of differentially expressed genes (DEGs) (compared with the H56 alone group) in the whole blood (WB) (**a**) and lymph nodes (LNs) (**d**) are shown as line plots. The Venn diagram depicts the numbers of unique and shared DEGs in WB (**b**) and LNs (**e**) at 24 h and 72 h. Volcano plots illustrate numbers of significantly downregulated (blue dots) and upregulated (red dots) DEGs for each adjuvant in WB (**c**) and LNs (**f**) over time.

Mice receiving H56 alone were used as a control group for the groups receiving H56 in combination with any of the four adjuvants. Differentially expressed genes (DEGs) were thus defined as those with a significant (adjusted *P* value < 0.05 FDR) change in expression in an adjuvant group compared with H56 alone. Alum did not induce any appreciable changes in the transcripts from WB, except for the 72 h time point, where 89 DEGs were detected, most of which were shared by other adjuvants (Fig. 1a,b). Although the kinetics and magnitude of the transcriptional changes varied widely among the groups, the other three adjuvants (CAF01, IC31 and GLA-SE) induced more dramatic changes in the WB transcriptional profile than Alum. GLA-SE was the only adjuvant that had already induced significant transcriptional changes (5,858 DEGs) at 6 h, with the highest number of DEGs (11,460 DEGs) at 72 h (Fig. 1a). CAF01 induced a later onset (24 h) of gene expression in WB (1,152 DEGs), and the highest number of significantly changed transcripts was observed at 72 h (4,490 DEGs) (Fig. 1a). The changes in the WB transcripts induced by IC31 were first observed and peaked at 24 h (3,191 DEGs) and decreased at 72 h (1,031 DEGs) (Fig. 1a). A higher proportion of downregulated transcripts was observed at the two early time points (6 h and/or 24 h), and greater numbers of upregulated transcripts were detected at 72 h for all adjuvants (Fig. 1c).

Next, we studied global changes in transcript profiles of dLNs in response to the different adjuvants. Overall, the transcriptional changes detected in dLNs were modest compared to those in WB. Alum induced limited changes in dLNs at 6 h. This number gradually increased at 24 and 72 h before peaking at 168 h (171 DEGs) (Fig. 1d). GLA-SE induced early changes in gene expression in dLNs at 6 h (1,787 DEGs), which increased at 24 h (9,440 DEGs) and 72 h (10,415 DEGs), followed by a decline at 168 h (5,420 DEGs), although the number of DEGs remained relatively high (Fig. 1d). Similar to the transcriptomic profile of WB, both CAF01 and IC31 induced much smaller numbers of DEGs in dLNs, with a later onset compared with GLA-SE. CAF01 caused changes in gene expression mainly at 24 h (460 DEGs) and only limited changes at 6 h, 72 h and 168 h (Fig. 1d). Similarly, the IC31-induced gene expression in dLNs peaked at 24 h (624 DEGs), decreased at 72 h, and then increased again at 168 h (Fig. 1d). Few DEGs were observed in dLNs of all adjuvant-treated groups, although at 24 h, GLA-SE shared 288 DEGs and 315 DEGs with IC31 and CAF01, respectively (Fig. 1e). The ratio between the up- and downregulated DEGs in dLNs showed similar trends for the different adjuvants across the different time points (Fig. 1f).

Overall, the adjuvants studied herein differed in terms of the magnitude and kinetics of the transcriptional changes induced in both WB and dLNs. In dLNs, GLA-SE induced the highest levels and the earliest onset of transcriptional changes, whereas both CAF01 and IC31 induced later and less detectable changes in the transcriptional profile compared with GLA-SE. Alum induced the most subtle transcriptional changes in both WB and dLNs.

### Gene functional patterns of WB revealed common and unique features of clinically tested adjuvants

**Figure 2:**
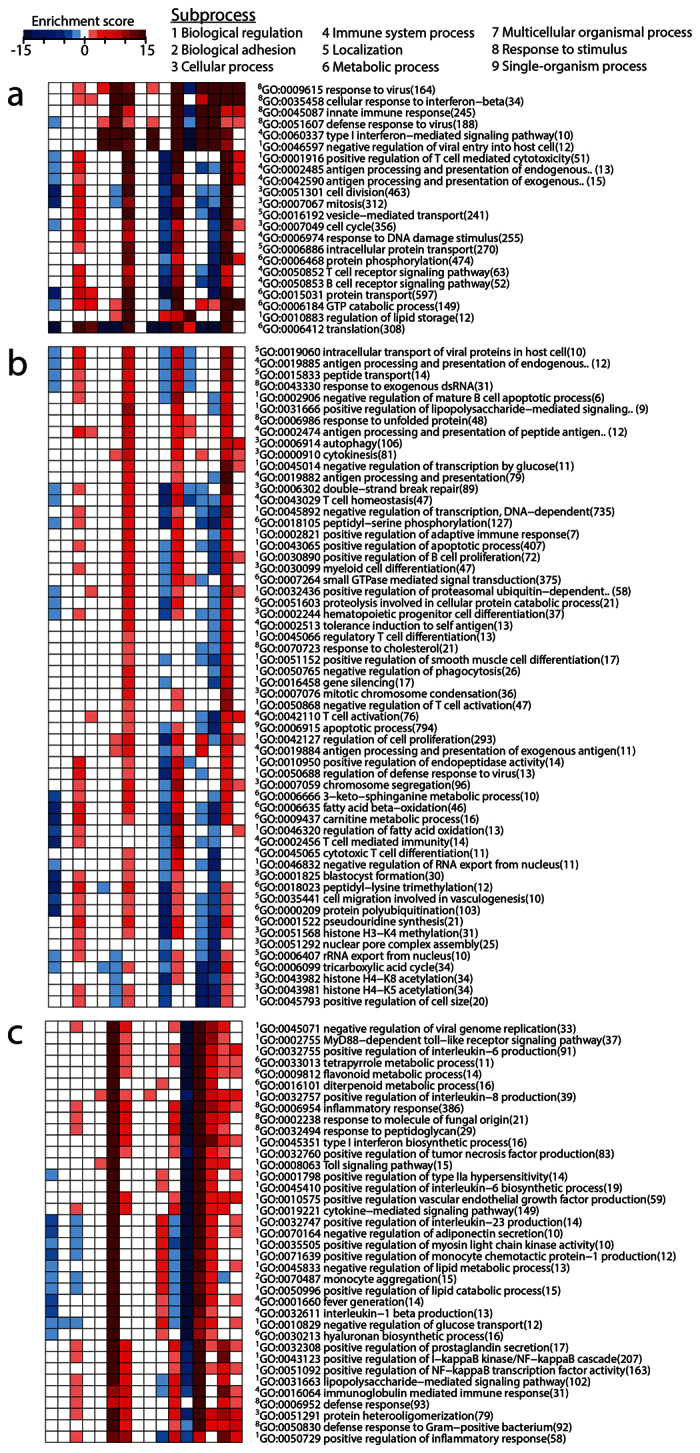
Analysis of gene ontology terms revealed shared and unique molecular signatures of vaccine adjuvants. A directional gene set enrichment analysis of the gene ontology (GO) terms in the DEGs was performed using the PIANO package. At 72 h, a group of GO terms was identified as commonly upregulated by all four adjuvants (**a**), and another group of GO terms was commonly upregulated by CAF01, IC31 and GLA-SE (**b**). A third group of GO terms was commonly upregulated by CAF01 and GLA-SE at early time points, whereas it was heavily downregulated by IC31 at 168 h (**c**). The heatmap shows the value of the enrichment score (−log10(enrichment P value)). The GO terms are color-coded to illustrate the direction of the gene expression changes in the majority of genes included in the respective GO term, i.e., red for upregulation and blue for downregulation.

To identify patterns of biological processes that were affected by the four adjuvants in WB, gene ontology (GO) enrichment mapping was performed for the DEGs of all adjuvants at the different time points. Three GO term groups were selected based on the clustering dendrograms, as explained in Materials and Methods. Distinct areas of enriched GO terms with upregulated DEGs were observed for all four adjuvants at 72 h (Supplementary Fig. 1). Grouping of these GO terms revealed that most belonged to cellular, metabolic and immune system processes, as well as to responses to stimuli (Fig. 2a). Another distinct group of GO terms belonging to biological regulation and metabolic and cellular processes was significantly enriched in the majority of the upregulated DEGs at 72 h in the CAF01, IC31 and GLA-SE groups, but to a lesser extent in the Alum group (Fig. 2b). Many of the GO terms that were commonly changed in WB (mainly at 72 h) by the three novel adjuvants were related to the initiation of adaptive immune responses (e.g., positive regulation of adaptive immune responses, positive regulation of B cell proliferation, and antigen processing and presentation).

Large numbers of GO terms were significantly enriched at early time points in the CAF01 (24 h and decreasing by 72 h) and GLA-SE groups (6 h and gradually decreasing by 168 h), with the majority of the DEGs being upregulated. These GO terms were not significantly enriched in the IC31 group at 6 h or 24 h, and they mainly contained downregulated DEGs at 72 h. The majority of these GO terms belonged to biological regulation and response to stimuli associated with innate and inflammatory functions, such as positive regulation of monocyte chemotactic protein-1 production (MCP-1) and positive regulation of interleukin (IL)-6 and IL-1β production (Fig. 2c).

### Induction of systemic responses of cytokines and chemokines by clinically tested adjuvants

**Figure 3:**
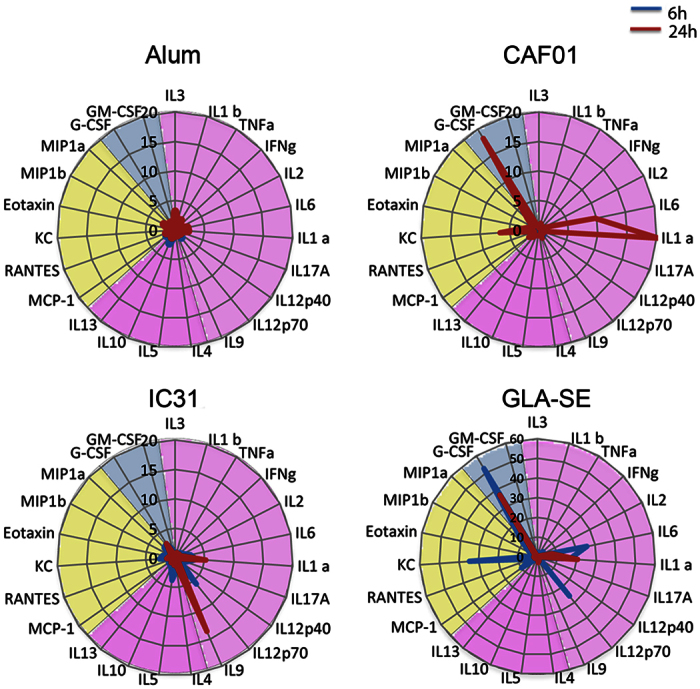
Induction of serum responses of cytokines and chemokines by clinically tested adjuvants. The radar plots show fold changes of cytokines and chemokines induced by different adjuvants over those of the H56 alone at 6 h (blue line) and 24 h (red line). The protein color codes are: cytokines (violet), chemokines (yellow) and growth factors (blue). Note the different scale for GLA-SE compared with the other adjuvant groups.

Next, we compared the patterns of cytokines and chemokines in sera of mice after administration of the adjuvants using a 23-plex luminex assay, including many known pro-and anti- inflammatory mediators. In line with the whole blood transcriptomics data, we observed the most subtle and greatest responses of cytokines and chemokines by Alum and GLA-SE, respectively (Fig. 3). GLA-SE induced elevated levels of the inflammatory cytokines IL-6 (fold change over H56 alone (FC) 25.61), IL12p70 (FC 25.73) and IL1α, (FC16.55) the neutrophil chemoattractant molecule KC (FC 34.72), the macrophage inflammatory protein MIP1-α (FC 10.23) and MCP-1 (FC 9.97) as well as granulocyte colony stimulating factor (G-CSF) (FC 52.52) already at 6 h with IL1α (FC 20.46) and G-CSF (FC 36.81) remaining elevated at 24 h. In line with the transriptomics data, IC31 and CAF01 induced delayed cytokine responses compared with GLA-SE. Thus, CAF01 induced increased levels of IL-6 (FC 9.75), IL1α (FC 20.31), KC (FC 6.62) and G-CSF (FC 18.18) at 24 h whereas IC31 induced much lower levels of IL1α (FC 5.24) and was the only adjuvant inducing high levels of IL-9 (FC 13.57). Taken together, these results, in line with the transcriptomics data, show that GLA-SE induces early and strong pro-inflammatory responses, and that CAF01 and IC31 mount delayed pro-inflammatory cytokine responses of smaller magnitude compared to those of GLA-SE.

### Co-expression analysis in WB revealed shared and unique features of clinically tested adjuvants

**Figure 4:**
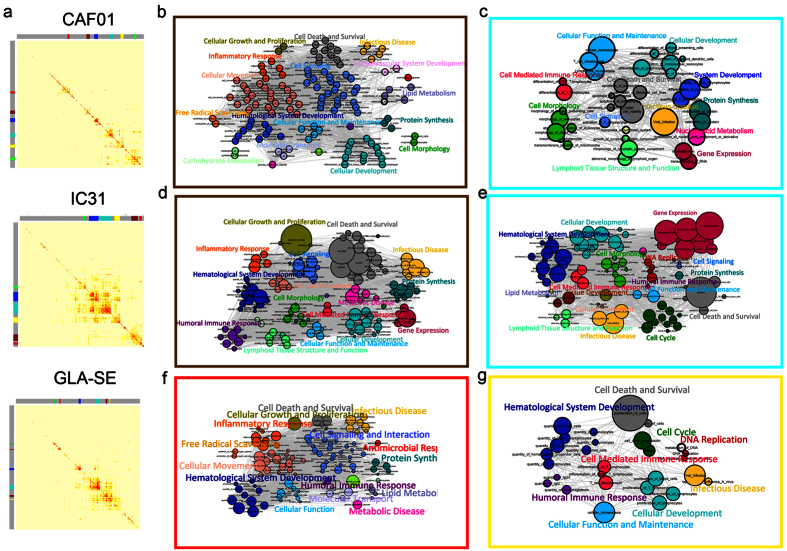
Co-expression analysis of DEGs identified common and unique biofunctions of vaccine adjuvants. Dendrogram from the gene co-expression analysis. Modules of significantly co-expressed genes were assigned different colors (**a**). Selection of the biofunction identified by IPA for the gene list of the brown and turquoise modules for CAF01 (**b** and **c**) and IC31 (**d** and **e**) as well as the red and yellow modules for GLA-SE (**f** and **g**). The size of each bubble is proportional to the number of DEG molecules involved in the given biofunction. Biofunctions belonging to the same category are color-coded.

Next, a weighted gene co-expression network analysis of DEGs across all time points for each adjuvant was conducted to identify the most highly affected gene modules (groups of highly co-expressed genes). Co-expression analyses utilize interaction patterns among significantly changed genes to reduce high-dimensional microarray datasets in an unbiased way. The co-expression analysis of DEGs in WB identified six different co-expressed modules for CAF01, IC31 or GLA-SE; each module was assigned a color code (Fig. 4a). The modules contained various numbers of genes, ranging from 77 genes (GLA-SE red module) to 566 genes (GLA-SE turquoise module). No co-expression module was identified for Alum due to its limited number of DEGs. Next, gene lists were generated from each module, and Ingenuity Pathway Analysis (IPA) was employed to identify enrichments in known biofunctions. The significantly changed biofunctions for each module and the associated DEGs are listed in Supplementary Table 1. For each adjuvant we decided to focus on those modules having most biofunctions associated with innate responses (CAF01; brown, Fig. 4b. GLA-SE; red, Fig. 4f) and induction of adaptive immunity (CAF01; turquoise, Fig. 4c. IC31; brown, Fig. 4d and turquoise, Fig. 4e. GLA-SE; yellow, Fig. 4g).

Whereas biofunctions associated with innate immune responses were scattered across different modules of the IC31 group (Supplementary Table 1), both GLA-SE and CAF01 had distinct modules containing molecules involved in innate inflammatory and phagocytosis responses (brown for CAF01 and red for GLA-SE, Figs 4b and f). For both adjuvants, the majority of the biofunctions were associated with cellular movement, such as leukocyte migration, chemotaxis of myeloid cells, chemotaxis of phagocytes and cell movement of neutrophils. Macrophage-inducible C-type lectin (Mincle) transcripts (Clec4e) were noted in many of the most highly significant innate biofunctions for both CAF01 (brown module) and GLA-SE (red module) (Supplementary Table 1). Mincle was first described as being induced upon lipopolysaccharide stimulation^18^ and has recently been reported as a key receptor for the CAF01^19^.

**Figure 5:**
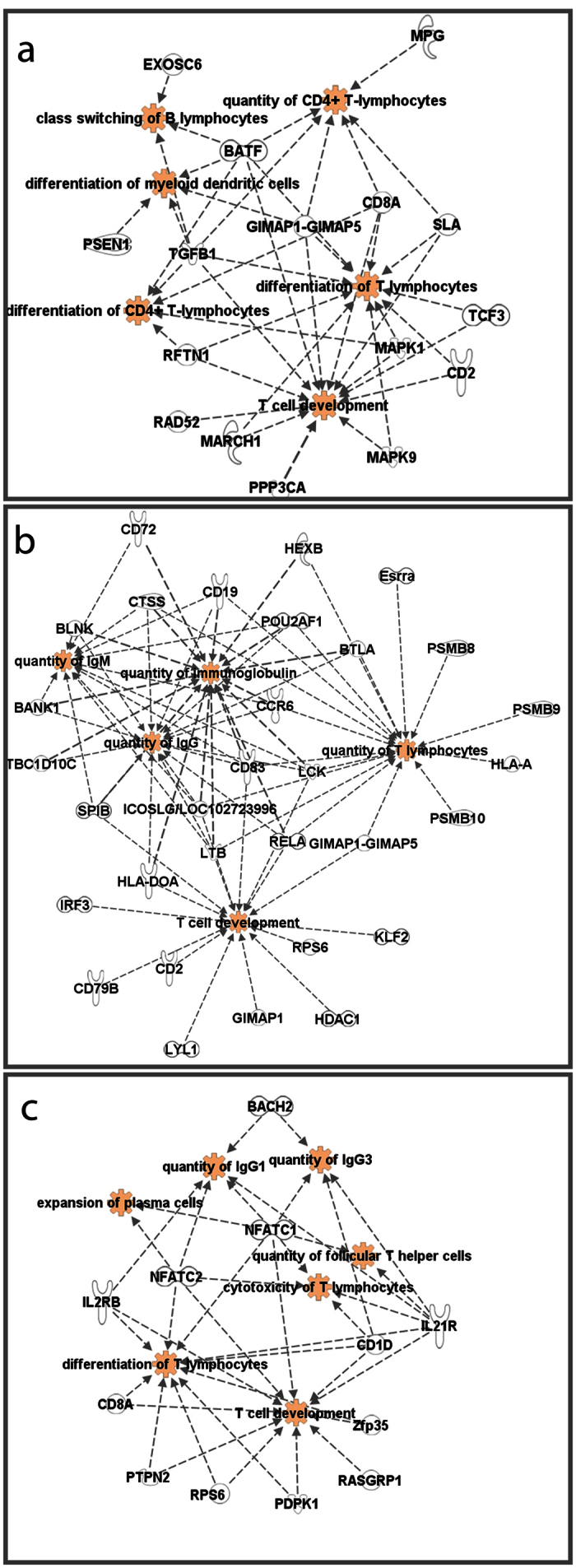
Co-expression analysis revealed adjuvant-specific molecules involved in adaptive immunity-related biofunctions. Relationship of the selected B and T cell-related biofunctions with the DEGs induced by CAF01 (**a**), IC31 (**b**) and GLA-SE (**c**) as identified by coexpression analysis. Biofunctions shown in red color. 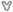 Cytokine/Growth factor, 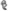 Enzyme, 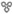 Kinase, 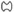 Transcriptional regulator, 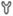 Transmembrane receptor, 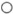 Other.

The brown module with the most highly significant biofunctions in the IC31 group included categories of biofunctions that are directly linked to adaptive immune responses, such as humoral immune response and cell-mediated immune response. Furthermore, many of the other categories, such as cellular development and hematological system development, contained biofunctions associated with the initiation of adaptive immune responses, including proliferation of lymphocytes and quantity of lymphocytes, respectively. Pou class 2-associating factor 1 (*Pou2af1*), a molecule shown to play an important role in multiple stages of B cell development and GC formation in mice upon immunization with T cell-dependent antigen^20^,^21^, was one of the transcripts involved in most biofunctions of adaptive immunity (Fig. 5b). Other biofunctions associated with the activation of adaptive immune responses and lymphocyte differentiation were detected in several other modules in the IC31 group, particularly the turquoise module (Fig. 4e and Supplementary Table 1).

For CAF01, biofunctions associated with lymphocyte activation were most abundant in the turquoise module, with the majority related to T cells and antigen-presenting cells (APCs) across different categories, such as differentiation of myeloid dendritic cells (DCs), differentiation of CD4^+^ T lymphocytes and class switching of B lymphocytes (Fig. 4c). Transcripts for the cytokine *Tgfb a*nd the transcription factor *Batf* were among the co-expressed molecules that were most commonly associated with the differentiation of APCs and lymphocytes in the CAF01 group (Fig. 5a). Batf is a transcription factor with important roles in the development of Th17, Th2 and TFH cells, as well as in the differentiation of Ab-producing B cells^22^.

For GLA-SE, most of the highly significant biofunctions associated with lymphocyte activation were detected in the yellow module. These biofunctions included differentiation of T lymphocytes, function of B lymphocytes and quantity of TFH cells (Fig. 5c and Supplementary Table 1). The biofunctions associated with lymphocyte activation and differentiation in the yellow module of GLA-SE in WB included transcripts of nuclear factor of activated T cells 1 and 2 (*Nfatc1* and *Nfatc2*) and IL-21 receptor (*Il21r*) (Fig. 5c), which have known functions in T cell activation, TFH activation and GC reactions.

Overall, co-expression analysis of DEGs in WB revealed that CAF01, IC31 and GLA-SE had distinct gene modules associated with biofunctions of adaptive immune responses, and that these gene modules contain molecules with documented roles in mounting characteristic T cell responses induced by each adjuvant.

### Co-expression and biofunction analysis of dLNs

The co-expression analysis of DEGs in dLNs identified distinct modules for IC31 and GLA-SE. However, due to the limited numbers of significant DEGs in dLNs in the Alum and CAF01 groups, the co-expression analysis for these adjuvants revealed no distinct modules. Two modules were defined for IC31 and 11 modules for GLA-SE, with variable numbers of transcripts ranging from 70 DEGs in the blue module for IC31 to 774 DEGs in the turquoise module for GLA-SE (Supplementary Table 2). The small blue module of IC31 contained molecules involved in immune cell trafficking, such as the accumulation of granulocytes and recruitment of leukocytes, as well as the initiation of adaptive (especially cell-mediated) immune responses, including the density and recruitment of T lymphocytes. The turquoise module of GLA-SE contained the most highly significant biofunctions, many of which were associated with infectious diseases, inflammatory responses and hematological system development and function. Other significantly changed biofunctions for GLA-SE associated with the initiation of immune responses following vaccination, included the cellular immune response, quantity of APCs, and quantity of IgG. A few other immune-related biofunctions were spread across the different modules of the GLA-SE group, including the quantity of IgG2a and development of TFH cells in the green-yellow module. The significantly changed biofunctions for each module and the associated DEGs in dLNs are listed in Supplementary Table 2.

### Adjuvant-mediated induction of TFH cell population in dLNs

**Figure 6:**
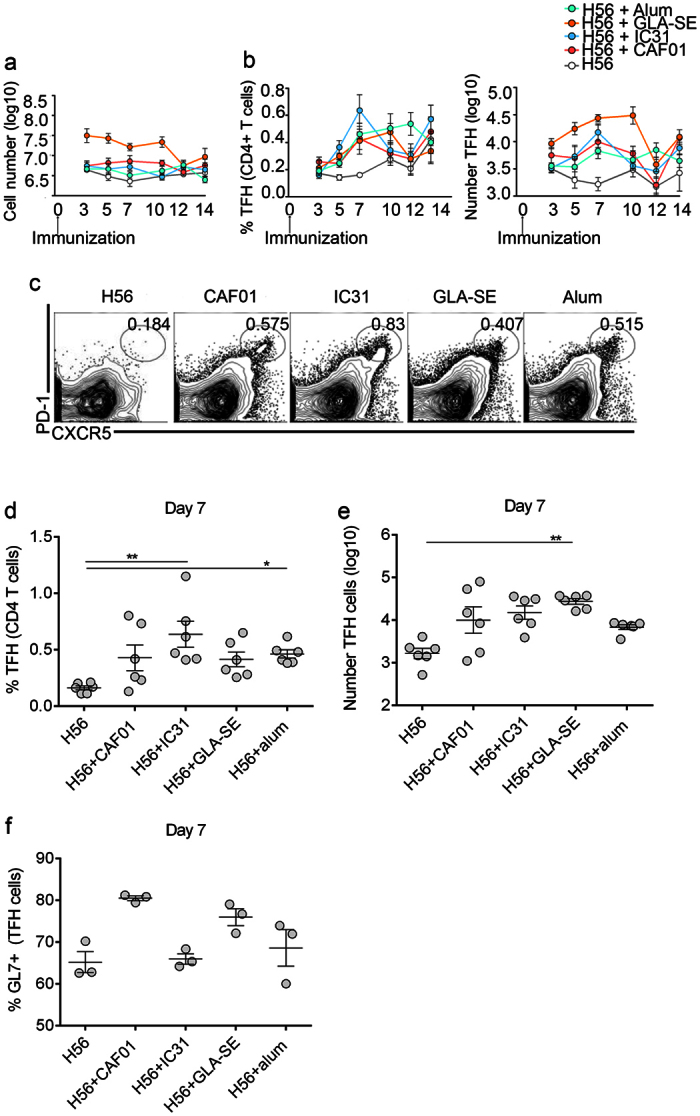
Induction of T follicular helper cell responses in draining LNs by vaccine adjuvants. Groups of mice were s.c. immunized with H56 alone or in combination with an adjuvant. Draining LNs were collected on days 3, 5, 7, 10, 12 and 14 after immunization, and the number of live cells was counted using the trypan blue exclusion assay (**a**). Frequency of T follicular helper (TFH) cells among the CD4 T cells and the total number of TFH cells were evaluated by flow cytometry (**b**). TFH cells were gated as PD1high CXCR5high, as shown by the representative plots (**c**). Graphs summarizing the frequency of TFH cells among the CD4 T cells (**d**) and the number of TFH cells on day 7 (**e**). The frequency of GL7+ TFH cells (**f**) on day 7. n = 5–6 mice at each time point, except for panel f (n = 3). The data from two independent experiments were pooled. The dots indicate individual values and are shown as means with error bars that represent the ±SEM. Significance was calculated using the Kruskal-Wallis test.

The unsupervised co-expression analysis described above identified adjuvant-specific gene modules that included molecules with known functions in TFH cells and/or the formation of GCs. This finding prompted us to study the impact of these human adjuvants on GC reactions and TFH cell responses in dLNs following immunization with H56 and the adjuvants. Mice were s.c.-immunized once with H56 alone or in combination with any of the four adjuvants. During the first 10 days a 10-fold expansion of the total dLN cell numbers was observed in the GLA-SE adjuvant-treated mice compared to the H56 group alone, whereas a more modest expansion ranging from 2- to 5-fold was induced by the other three adjuvants over the same time period (Fig. 6a). This suggests a very rapid and broad immune induction in the local dLN by GLA-SE, which was also indicated by the massive number of DEGs in both dLNs and blood within the first 72 h after immunization (Fig. 1). The TFH cells in dLNs on days 3, 5, 7, 10, 12 and 14 after immunization (Fig. 6b) were studied by evaluating the frequencies of CXCR5^hi^, PD-1^hi^ CD4^+^ T cells (Fig. 6c). The frequencies and numbers of TFH cells remained low in the H56-immunized animals throughout the study. In contrast, all of the tested adjuvants enhanced TFH cell frequencies, and the most increase in frequencies of TFH cells relative to CD4^+^ T cells compared with H56 alone were observed 7 days after immunization. Similarly, adjuvant induced increase in total number of TFH cells was observed on day 7 compared with H56 alone, followed by a dramatic decrease on day 10 for IC31 and day 12 for GLA-SE and CAF01 (Fig. 6b). The mean frequencies of TFH cells were comparable among the different adjuvant groups on day 7, although the intra-group variations ensured that only the Alum and IC31 groups showed a statistically significant increase in TFH frequency compared to H56 alone (Fig. 6d). Higher numbers of TFH cells were only detected in the GLA-SE group (*P* < 0.01), but with a trend toward higher numbers in the other three adjuvant groups also when compared with the H56 alone group, reflecting a difference in total cell numbers in dLNs among the different adjuvant groups (Fig. 6e). Fully differentiated GC TFH cells have been shown to express GL7 (CXCR5^+^ GL7^+^), in contrast to the TFH (CXCR5^+^ GL7^−^) cells located outside of the GC^23^. High proportion of TFH cells in the dLNs of the different adjuvant groups expressed GL7, indicating that most of the cells falling within our TFH gate were fully differentiated with the highest frequency of GL7 TFH cells observed in CAF01 and GLA-SE groups on day 7 (Fig. 6f).

Taken together, these results show that all of the tested adjuvants enhanced the frequencies of TFH cells compared with H56 alone. Although a big variation in the TFH induction kinetics was seen among the adjuvants, no significant difference in frequencies was observed. GLA-SE induced the highest absolute numbers of TFH cells within the sampling period reflecting a significantly larger expansion of the dLN after vaccination when compared to the other adjuvants. The major proportion of adjuvant-induced TFH cells was also shown to express GL7 following immunization, indicating that these cells had completely differentiated into TFH cells.

### Clinically tested adjuvants induced different GC reactions

**Figure 7:**
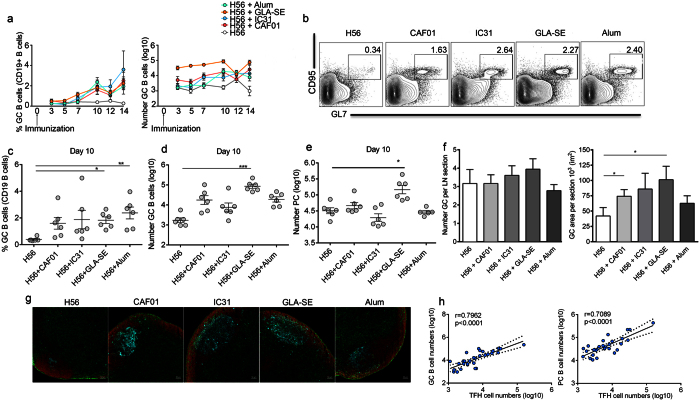
Germinal center reactions in draining LNs induced by adjuvants. Mice were s.c. immunized with H56 alone or in combination with the indicated adjuvants. Draining LNs (dLNs) were collected on days 3, 5, 7, 10, 12 and 14 after immunization, and the frequency of germinal center (GC) cells among the CD19+ B cells and the total number of GC B cells were evaluated by flow cytometry (**a**). Germinal center (GC) B cells were gated as GL7+ CD95+ among the CD19+ B cells, as shown by the representative plots (**b**). Graphs summarizing the frequency (**c**) and total numbers (**d**) of GC B cells as well as total numbers (**e**) of cells with the plasma cell (PC) phenotype CD138+ B220int/low on day 10 after immunization. Column plots showing the number of GCs and size of the GC area per section in μm^2^ counted from 18 dLNs sections from each group (**f**). One representative section of a dLN showing immunohistochemical staining for GL7 (blue), B220 (red), and CD4 (green) on day 10 after immunization (**g**). The number of GCs per section was determined by microscopy, and the surface (μm^2^) of the GC was measured using ImageJ 1.49v software. Correlations between the numbers of TFH cells and GC B cells on day 10 for all groups are shown on the left, and the numbers of TFH cells and PC cells on day 10 are shown on the right (**h**). The results are expressed as the means ± SEM and significance was calculated using the Kruskal-Wallis test. Correlations were assessed using Spearman’s rank test. *P < 0.05, **P < 0.01, ***P < 0.001.

TFH cells induce and regulate GC formation as well as B cell maturation and differentiation culminating in high-affinity Ab responses. We investigated the induction of GC B cells in dLNs on days 3, 5, 7, 10, 12 and 14 after immunization, i.e., the same time points used in the TFH cell experiment described above. Although the induction of TFH cells peaked 7 days after immunization for all the adjuvant groups, the differentiation of B cells into GC B cells peaked on day 10 in terms of both frequency and numbers (Fig. 7a). GC B cells were gated as GL7^+^ and CD95^+^ among the CD19^+^ B cells and represented a clearly distinct population on day 10 in all of the adjuvant groups (Fig. 7b). Significantly higher frequencies of GC B cells were observed in the Alum (*P* < 0.01) and GLA-SE (*P* < 0.05) groups compared with the group receiving H56 alone, whereas more variation was observed in the CAF01 and IC31 groups (Fig. 7c). The greatest increase in the total number of GC B cells was observed in the GLA-SE (*P* < 0.001) group compared with the H56 group (Fig. 7d). Furthermore, GLA-SE (*P* < 0.05) was the only adjuvant that enhanced the number of cells with the PC phenotype (CD138^+^ B220^int/low^) at the early time points (Fig. 7e). Next, we examined the formation of GCs in dLNs on day 10 after immunization using immunohistochemistry with antibodies against GL7 (blue), B220 (red) and CD4 (green) (Fig. 7g). Immunohistochemical staining revealed that although the number of GCs in dLNs did not differ significantly among the immunized groups, all of the mice immunized with adjuvant plus H56 showed an increased GC size compared with the H56 alone-immunized mice (Fig. 7f). Statistically significant differences were only observed for the CAF01 and GLA-SE groups (*P* < 0.05) (Fig. 7f). The induction of GC B cells and PCs strongly correlated with the induction of TFH cells (Fig. 6h).

Although GC formation lagged behind TFH cell induction in mouse dLNs following immunization with the H56 antigen and the adjuvants, these results reveal that the two processes were correlated. Interestingly, all of the tested adjuvants enhanced GC formation, as demonstrated by the GC size and GC B cell frequency. However, differences were observed in the adjuvants’ ability to enhance total numbers of GC B cells and early PC phenotypes in the dLNs.

## Discussion

Systems biology approaches have recently been employed to define early signatures of immune responses induced by different human vaccines and adjuvants (reviewed in refs 5 and 6). However, direct comparative analyses of different human adjuvants have been hampered by a lack of uniformity in experimental design and data analysis, combined with the unavailability of proprietary adjuvants. Herein, we report the early molecular signatures in mouse WB and dLNs of three clinically tested vaccine adjuvants, namely CAF01, IC31 and GLA-SE, along with Alum.

Wide variation in transcriptional changes in both magnitude and kinetics was observed among these adjuvants, with Alum and GLA-SE inducing the smallest and largest numbers of transcriptional changes, respectively, in both WB and dLNs. These results are in accordance with a previous report showing that immunization with Alum induced only limited transcriptional changes in WB and dLNs when compared with GLA-SE^24^. CAF01 and IC31, on the other hand, induced fewer transcriptional changes in WB and dLN compared to GLA-SE. CAF01 and IC31 were previously shown to form a depot at the injection site with only slightly increased activity in the dLN over a prolonged time period^25^,^26^, and therefore it is plausible that a higher magnitude of gene expression could be observed at their injection site. The massive transcriptional changes induced by GLA-SE both in WB and in dLNs correlated with a much higher expansion of the total cell number in the dLNs of the GLA-SE group compared with those of the other three adjuvants. The differences between the CAF01 and IC31 groups and the GLA-SE group in terms of the overall kinetics and magnitude of *in vivo* transcriptional changes presumably reflect the different cell subpopulations targeted by the adjuvants and the *in vivo* kinetics of the adjuvants. Recently, GLA-SE was found to induce neutrophils to engulf antigen at injection site and travel to subcapsular lymphatics as well as T cell areas of the dLNs within 24 hours where they produced high amounts of IFN-γ^27^. Further, GLA-SE was shown to dramatically enhance the number of activated CD11c^+^ GR1^+^ myeloid DCs in mouse LNs within 24 h after immunization^24^ whereas CAF01 and IC31 were found to target a minute number of vaccine-associated DCs *in vivo* over a period of 2 weeks in mice^25^,^26^,^28^. These observations could, at least in part, explain the limited transcriptional changes induced by IC31 and CAF01 in both WB and dLNs as compared to GLA-SE. Furthermore, the early onset of transcriptional changes induced by GLA-SE in the dLNs (6 h) is in line with a previous report^24^. Nevertheless, the massive cell infiltration and early transcriptional changes associated with inflammatory responses induced by GLA-SE returned to the baseline levels within 7 days after administration. It is noteworthy that all of these adjuvants have previously been reported to be well tolerated with acceptable safety profiles in human trials.

Co-expression analyses have been widely used to elucidate the molecular mechanisms of various immunological properties, including human blood transcriptome modules, following vaccination^29^,^30^. The co-expression analysis of DEGs in WB presented herein revealed both common and unique biofunctional signatures for the three novel adjuvants. Both CAF01 and GLA-SE induced modules with biofunctions that were related to innate immune responses, such as the recruitment of various innate cells, chemotaxis and phagocytosis. IC31, on the other hand, did not exhibit a distinct module of DEGs associated with innate immune responses, and the corresponding DEGs were scattered across different modules. This is in accord with the observation that IC31 was the only adjuvant among the three without highly upregulated DEGs belonging to GO terms for innate inflammatory responses. The observed limited pro-inflammatory transcript expression is in line with a previous report that IC31 mainly targets monocyte-derived DCs by increasing the level of IFN-β and limiting the production of the pro-inflammatory cytokines TNF-α and IL-6^31^.

Gene modules in WB that were mainly associated with adaptive immune-related biofunctions were identified for all four adjuvants. An extensive immunological comparison of the same four adjuvants was recently reported by Knudsen *et al*. revealing that the adjuvants induced different CD4+ T cell polarization as well as IgG Ab subclass switching independent of the choice of antigen^7^. Our analyses of the main molecules involved in biofunctions related to adaptive immunity revealed intriguing differences that might explain the observed differences in B and T cell responses induced by the different adjuvants.

Interestingly, our co-expression analysis identified adjuvant-specific WB gene modules containing genes with known function in GC TFH cell-mediated B cell responses, including *Nfatc1, Nfatc2* and *IL21R* (induced by GLA-SE)^32^,^33^,^34^, *Batf* (induced by CAF01)^22^ and *Pou2af1* (induced by IC31)^20^,^21^. All four adjuvants tested herein induced elevated TFH cell differentiation in dLNs 7 days after co-administration with H56, when compared to H56 alone. This was followed by induction of GC B cells peaking on day 10. It should be noted that the small percentages of TFH and GC B cells detected in the dLN of the H56 alone group support our previous findings in which H56 alone was found immunogenic, albeit its immunogenicity was dramatically enhanced when combined with CAF01, GLA-SE and IC31^7^. GLA-SE induced the highest numbers of TFH cells and GC B cells at day 10, which agrees with a recent report on the ability of GLA-SE in induction of a strong Ab response already after one immunization, whereas IC31, CAF01 and Alum induced significant Ab responses only after two or three immunizations^7^. The squalene-based adjuvant, MF59, has been reported to reach the dLNs within 6 h following co-injection with Ag where it promoted retention of intact Ag entrapped in immune complexes within macrophages translocating the vaccine to the follicular DCs pivotal in initiating the GC reaction^35^,^36^. The close resemblance between the SE part of GLA-SE and MF59 suggests that similar processes could be mediated by the two adjuvants. Thus, it is likely that the broad activation of dLN-resident cells in response to GLA-SE may play a role in the massive dLN expansion, the strong transcriptional changes and the early induction of GC B cells and PCs reported herein and the subsequent Ab response as previously reported^7^. Our data also support the notion that the number of TFH cells strongly correlates with the adjuvant-induced B cell responses^37^.

It has previously been reported that IC31 and CAF01 induce durable and potent characteristic CD4+ T cell responses in mice and humans^7^,^8^,^38^,^39^,^40^. Interestingly, *Batf* and *Tgfb*, with documented roles in Th17 differentiation, were among the co-expressed molecules within adaptive immune-related biofunctions for the Th17-inducing adjuvant CAF01^41^,^42^. Further, genes involved in Th1 differentiation such as *Pou2af1* and *Btla,* were among the co-expressed molecules for the Th1-tilting adjuvant IC31^43^. Nevertheless, we were unable to perform a statistical correlation analysis on the early molecular signatures and immunological readouts due to the lack of stratified immune responses in inbred mice.

In line with other publications, we observed subtle transcriptional changes induced by Alum compared with the other tested adjuvants, despite the ability of Alum in mounting a potent antibody response and the induction of TFH and GC B cell responses in the draining lymph nodes of mice^24^,^44^,^45^,^46^. Mode of action of Alum is incompletely understood and suggested mechanisms of action include increased Ag uptake, recruitment of innate inflammatory cells, such as neutrophils, eosinophils, inflammatory monocytes, myeloid DCs and plasmacytoid DCs to the site of injection, and subsequent activation of innate immune responses through various danger signals^47^,^48^. Although our systems biology analysis of Alum induced response presented herein add to the growing body of evidence in the literature on the mode of action of Alum, further studies are needed.

In summary, the head-to-head comparative transcriptomics and systems biology analyses of clinical-grade adjuvants reported herein have revealed gene expression profiles, biological pathways, biofunctions and gene modules in mouse WB and dLNs that are shared by or exclusive to the different adjuvants. We could also identify characteristic blood gene modules that were highly enriched for adaptive immune-related biofunctions, including molecules with documented roles in TFH and GC responses. These observations were supported by immunological analyses showing that, although with different magnitude, all groups receiving adjuvanted H56 showed enhanced TFH and GC responses in dLNs, compared with the H56 antigen alone. These results provide new insights into the mechanisms of action of three clinically tested adjuvants, along with Alum, and may inform rational development of new adjuvants for human use.

## Materials and Methods

### Animals

Six- to eight-week-old female CB6F1/OlaHsd mice (Harlan Laboratories, The Netherlands) were housed in ventilated cages with free access to food and water. All animals were housed under standardized pathogen-free conditions at the Experimental Biomedicine Animal Facility, University of Gothenburg.

### Ethics Statement

The use of mice was performed in accordance with the regulations set forth by the Ethical Committee for Animal Experimentation in Gothenburg, Sweden and in accordance with European Community Directive 86/609. All the techniques and procedures were refined to provide for maximum comfort and minimal stress to mice. Experiments performed were approved by the Ethical Committee for Animal Experimentation in Gothenburg, Sweden under license 64/2015.

### Adjuvants and antigen

The effects of the adjuvants were evaluated with 5 μg of clinical-grade *Mycobacterium tuberculosis* H56 fusion protein provided by Statens Serum Institute (SSI, Denmark). The following clinical-grade adjuvants were tested: Alhydrogel 2% (hereafter called Alum) provided by Brenntag Biosector (Denmark), CAF01 from SSI, IC31 from Valneva Austria GmbH (Austria), and GLA-SE from Infectious Disease Research Institute (Seattle, WA, USA). The adjuvants and antigens were combined strictly according to the manufacturers’ recommendations. Alum was diluted to 3.4 mg/ml of aluminum in saline. Then, H56 in saline was added to Alum at a 1:1 ratio (v/v) and rotated at 4 °C overnight for adsorption. CAF01 was mixed with H56 in Tris buffer (10 mM, pH 7.4) at a 1:1 ratio (v/v) and gently vortexed. IC31 was mixed with H56 in phosphate-buffered saline (PBS) such that the final concentration of each dose was 100 nmol KLK/4 nmol ODN1a, and then the mixture was gently inverted several times to ensure a homogeneous suspension. GLA-SE was vortexed with H56 in PBS to obtain a final concentration of 5 μg/dose. All vaccines were used within 2 h of final preparation.

### Study design and organ collection

Mice were lightly anesthetized with isoflurane (Baxter Healthcare) before immunization with H56 with or without adjuvant, as described above. Each mouse received a total volume of 100 μl of the vaccine mixture injected s.c. on both sides of the base of the tail. Each group included 28 mice. A group of non-immunized mice (n = 7) were sacrificed and served as a naïve control group (time point 0 h). Seven mice from each group were sacrificed at each of the four time points (6 h, 24 h, 72 h and 168 h) after immunization. WB was collected from the axillary plexus in PAXgene RNA stabilizer (Qiagen GmbH, Germany) such that the final ratio of stabilizer to blood was maintained at 2.76, as recommended by the manufacturer. Immediately after bleeding, the mice were sacrificed by cervical dislocation and the inguinal LNs were harvested in 1 ml of RNAlater (Qiagen).

In another set of experiments, groups of mice were immunized with the vaccine formulations described above. dLNs were collected 3, 5, 7, 10, 12 and 14 days after primary immunization to evaluate effects of the different vaccine formulations and immunization schemes on TFH cells and humoral responses.

### RNA extraction

RNA was extracted from dLNs and WB using the RNeasy Mini QIAcube kit (Qiagen) and PAXgene kit (Qiagen), respectively, according to the manufacturer’s instructions. Briefly, dLNs were homogenized in 350 μl of RLT buffer with metal beads in a TissueLyser II for 2 min. Further homogenization was performed by adding the samples to QIAshredder columns, followed by centrifugation at 14,000 rpm for 2 min. The samples, DNase from an RNase-free DNase kit (Qiagen), 70% ethanol and all the necessary reagents from the appropriate RNA extraction kits were then added to the QIAcube, according to the manufacturer’s protocol. The extracted RNA was kept on ice upon extraction, followed by an assessment of the RNA concentrations using a Nanodrop spectrophotometer (Thermo Scientific, USA). All the samples were examined on an Agilent 2200 TapeStation (Agilent Technologies, USA) to check the quality by measuring the RNA integrity number (RIN). High-quality RNA samples, which were defined as having a 260/280 ratio of approximately 2 and an RIN >8, were used in downstream applications.

### Whole-genome microarray analysis

High-quality RNA samples from WB and dLNs were subjected to a whole-genome microarray analysis on an Agilent platform (Agilent Technologies). The RNA was labeled with a Fluorescent Linear Amplification Kit according to manufacturer’s instructions. The quantity and labeling efficiency were verified before the samples were hybridized to whole-genome 8 × 60 k mouse expression arrays, which were scanned at 5 μm using an Agilent scanner. Image analysis was performed with Feature Extraction software (version 11.5.1.1, Agilent Technologies) to generate raw microarray data.

### Microarray data acquisition and analysis

Microarray data were pre-processed and normalized using the limma package for the R suite. The raw data were first corrected for background using the “normexp” method. Background-corrected signals were normalized together using the quantile method. The normalized gene signals were further evaluated for differential gene expression using the moderate Student’s *t*-test to compare the different groups with the control group at each time point, and the *P* values were further adjusted for multiple testing using the Benjamini-Hochberg method. Changes in expression induced by the adjuvants were calculated by dividing the log_2_ expression value of each individual adjuvant-treated group by the log_2_ expression value of the group receiving H56 alone at any given time point. A directional gene set GO enrichment analysis was performed using the PIANO package^49^. The results were plotted as a heatmap of the enrichment score (−log_10_ (enrichment *P* value)). To identify global functional profiles, GO terms were traced back to their corresponding main GO hierarchy. GO relationships were retrieved from go-basic.obo (released November 10, 2014), and the different levels of the GOs were generated using a custom script. Differential gene expression (adj *P* value < 0.05) was further used to identify concerted responses using a gene co-expression analysis method called weighted gene co-expression analysis^50^. This analysis was performed in the R suite using the R package WGCNA^51^. IPA software (Qiagen) was used to evaluate biofunctions associated with gene lists derived from co-expression analyses. Visualization of the selected co-expressed modules was performed with Cytoscape software^52^. The complete set of the microarray data was deposited in NCBI’s Gene Expression Omnibus and are accessible through GEO Series accession number GSE85339 (http://www.ncbi.nlm.nih.gov/geo/query/acc.cgi?acc=GSE85339).

### Multiparameter flow cytometric analysis

Inguinal LN cells were washed with PBS and stained with an amine-reactive viability dye (LIVE/DEAD Yellow, Invitrogen). For the T cell Ab panel, cells were stained with purified rat anti-CXCR5 Ab (2G8, BD Pharmingen) for 1 h at 4 °C, followed by a goat anti-rat biotin Ab (Jackson ImmunoResearch) for 30 min at 4 °C. Surface staining was performed together with streptavidin (Biolegend) labeling at 4 °C for 30 min using the following antibodies: CD3 (17A2), CD4 (RM4-5), PD-1 (29F.1A12), CD44 (BD Pharmingen), CD19 (6D5), CD138 (281-2), B220 (RA3-6B2), GL7 (GL7), and CD95 (Jo2, BD Pharmingen). All antibodies were from Biolegend unless otherwise indicated. Samples were acquired on an LSR II (BD Biosciences) flow cytometer. Compensation was performed using compensation beads labeled with a single Ab (UltraCompBeads, eBioscience). The data were analyzed using FlowJo version 9.2 (TreeStar Inc.). Lymphocytes were defined by FSC/SSC, and doublets were excluded by FSC-A/FSC-H gating. Live cells were gated as negative for Yellow Live/Dead staining, and CD4 T cells were defined as CD3^+^, CD19^−^, and CD4^+^. B cells were categorized as distinct subsets of GC B cells (CD3^−^, CD19^+^, CD95^+^, and GL-7^+^) or PCs (CD3^−^, B220^int/low^, and CD138^+^).

### Immunohistochemistry

Inguinal LNs (n = 3) were collected in Histocon (HistoLab) 10 days after immunization, embedded in Tissue-Tek OCT (Sakura), snap-frozen in isopentane cooled in liquid N_2_ and stored at −80 °C until they were cut into 7 μm sections. The sections were fixed in acetone and stored at −20 °C until staining. Three sections of different depths from each mouse were stained. Slides were blocked with 10% goat serum and stained overnight with biotinylated anti-B220 (RA3-6B2, BD Pharmingen), anti-CD4-FITC (L3T4, eBioscience) and anti-GL-7-eFluor660 (GL-7, eBioscience) at 4 °C, followed by a 1-h incubation with streptavidin-Alexa Fluor 594 (Life Technologies) at room temperature. Slides were mounted with Fluorescent Mounting Medium (Dako) and examined with a Zeiss Axio Imager.Z2 microscope. Images were acquired with Zeiss Zen Pro software. The number of GCs per section was counted under the microscope, and the GC area in each section was quantified using ImageJ 1.49 v software (NIH, USA).

### Assessment of cytokine and chemokines in serum

Serum samples were collected at 6 h and 24 h and stored at −20 °C until analyzed with a Bio-Plex Pro Mouse Cytokine 23-plex Assay (Biorad). The luminex assay was performed according to the manufacturer’s protocol and run on MagPix equipment (Invitrogen). Fold change (FC) calculation of proteins induced by the different adjuvants were calculated by dividing the mean values of each protein in different adjuvant groups by the mean values of those of the H56 alone group.

### Statistical analyses

Statistical analyses were performed using GraphPad Prism 5.0 (GraphPad Software). A Kruskal-Wallis post-test was used with a 95% confidence interval to compare multiple groups. Spearman’s correlation analysis was performed, and *P* values < 0.05 were considered significant.

## Additional Information

**How to cite this article**: Olafsdottir, T. A. *et al*. Comparative Systems Analyses Reveal Molecular Signatures of Clinically tested Vaccine Adjuvants. *Sci. Rep.* **6**, 39097; doi: 10.1038/srep39097 (2016).

**Publisher's note:** Springer Nature remains neutral with regard to jurisdictional claims in published maps and institutional affiliations.

## Supplementary Material

Supplementary Table 1

Supplementary Table 2

Supplementary Figure 1

## References

[b1] Guy B. The perfect mix: recent progress in adjuvant research. Nat Rev Microbiol 5, 505–517 (2007). 17558426 10.1038/nrmicro1681

[b2] Coffman R. L., Sher A. & Seder R. A. Vaccine adjuvants: putting innate immunity to work. Immunity 33, 492–503 (2010). 21029960 10.1016/j.immuni.2010.10.002 PMC3420356

[b3] Haralambieva I. H. . Genome-wide characterization of transcriptional patterns in high and low antibody responders to rubella vaccination. PLoS One 8, e62149, doi: 10.1371/journal.pone.0062149 (2013). 23658707 PMC3641062

[b4] Rappuoli R., Mandl C. W., Black S. & De Gregorio E. Vaccines for the twenty-first century society. Nat Rev Immunol 11, 865–872, doi: 10.1038/nri3085 (2011). 22051890 PMC7098427

[b5] Pulendran B. Systems vaccinology: probing humanity’s diverse immune systems with vaccines. Proc Natl Acad Sci USA 111, 12300–12306, doi: 10.1073/pnas.1400476111 (2014). 25136102 PMC4151766

[b6] Olafsdottir T., Lindqvist M. & Harandi A. M. Molecular signatures of vaccine adjuvants. Vaccine 5302–5307, doi: 10.1016/j.vaccine.2015.04.099 (2015). 25989447

[b7] Knudsen N. P. . Different human vaccine adjuvants promote distinct antigen-independent immunological signatures tailored to different pathogens. Sci Rep 6, 19570, doi: 10.1038/srep19570 (2016). 26791076 PMC4726129

[b8] Lindenstrom T. . Tuberculosis subunit vaccination provides long-term protective immunity characterized by multifunctional CD4 memory T cells. J Immunol 182, 8047–8055, doi: 10.4049/jimmunol.0801592 (2009). 19494330

[b9] Agger E. M. . Cationic liposomes formulated with synthetic mycobacterial cordfactor (CAF01): a versatile adjuvant for vaccines with different immunological requirements. PLoS One 3, e3116, doi: 10.1371/journal.pone.0003116 (2008). 18776936 PMC2525815

[b10] Lingnau K., Riedl K. & von Gabain A. IC31 and IC30, novel types of vaccine adjuvant based on peptide delivery systems. Expert Rev Vaccines 6, 741–746 (2007). 17931154 10.1586/14760584.6.5.741

[b11] Boughammoura S., Kessabi K., Chouchene L. & Messaoudi I. Effects of cadmium and high temperature on some parameters of calcium metabolism in the killifish (Aphanius fasciatus). Biological trace element research 154, 73–80, doi: 10.1007/s12011-013-9714-8 (2013). 23749477

[b12] Marrack P., McKee A. S. & Munks M. W. Towards an understanding of the adjuvant action of aluminium. Nat Rev Immunol 9, 287–293, doi: 10.1038/nri2510 (2009). 19247370 PMC3147301

[b13] Kool M., Fierens K. & Lambrecht B. N. Alum adjuvant: some of the tricks of the oldest adjuvant. J Med Microbiol 61, 927–934, doi: 10.1099/jmm.0.038943-0 (2012). 22174375

[b14] Aagaard C. . A multistage tuberculosis vaccine that confers efficient protection before and after exposure. Nat Med 17, 189–194, doi: 10.1038/nm.2285 (2011). 21258338

[b15] Lin P. L. . The multistage vaccine H56 boosts the effects of BCG to protect cynomolgus macaques against active tuberculosis and reactivation of latent Mycobacterium tuberculosis infection. J Clin Invest 122, 303–314, doi: 10.1172/JCI46252 (2012). 22133873 PMC3248283

[b16] Billeskov R. . Testing the H56 Vaccine Delivered in 4 Different Adjuvants as a BCG-Booster in a Non-Human Primate Model of Tuberculosis. PLoS One 11, e0161217, doi: 10.1371/journal.pone.0161217 (2016). 27525651 PMC4985151

[b17] Luabeya A. K. . First-in-human trial of the post-exposure tuberculosis vaccine H56:IC31 in Mycobacterium tuberculosis infected and non-infected healthy adults. Vaccine 33, 4130–4140, doi: 10.1016/j.vaccine.2015.06.051 (2015). 26095509

[b18] Matsumoto M. . A novel LPS-inducible C-type lectin is a transcriptional target of NF-IL6 in macrophages. J Immunol 163, 5039–5048 (1999). 10528209

[b19] Schoenen H. . Cutting edge: Mincle is essential for recognition and adjuvanticity of the mycobacterial cord factor and its synthetic analog trehalose-dibehenate. J Immunol 184, 2756–2760, doi: 10.4049/jimmunol.0904013 (2010). 20164423 PMC3442336

[b20] Kim U. . The B-cell-specific transcription coactivator OCA-B/OBF-1/Bob-1 is essential for normal production of immunoglobulin isotypes. Nature 383, 542–547, doi: 10.1038/383542a0 (1996). 8849728

[b21] Schubart D. B., Rolink A., Kosco-Vilbois M. H., Botteri F. & Matthias P. B-cell-specific coactivator OBF-1/OCA-B/Bob1 required for immune response and germinal centre formation. Nature 383, 538–542, doi: 10.1038/383538a0 (1996). 8849727

[b22] Betz B. C. . Batf coordinates multiple aspects of B and T cell function required for normal antibody responses. J Exp Med 207, 933–942, doi: 10.1084/jem.20091548 (2010). 20421391 PMC2867277

[b23] Yusuf I. . Germinal center T follicular helper cell IL-4 production is dependent on signaling lymphocytic activation molecule receptor (CD150). J Immunol 185, 190–202, doi: 10.4049/jimmunol.0903505 (2010). 20525889 PMC2913439

[b24] Lambert S. L. . Molecular and cellular response profiles induced by the TLR4 agonist-based adjuvant Glucopyranosyl Lipid A. PLoS One 7, e51618, doi: 10.1371/journal.pone.0051618 (2012). 23284726 PMC3532059

[b25] Kamath A. T. . Protective anti-mycobacterial T cell responses through exquisite *in vivo* activation of vaccine-targeted dendritic cells. Eur J Immunol 38, 1247–1256 (2008). 18412160 10.1002/eji.200737889

[b26] Kamath A. T. . A liposome-based mycobacterial vaccine induces potent adult and neonatal multifunctional T cells through the exquisite targeting of dendritic cells. PLoS One 4, e5771, doi: 10.1371/journal.pone.0005771 (2009). 19492047 PMC2685976

[b27] Desbien A. L. . Squalene emulsion potentiates the adjuvant activity of the TLR4 agonist, GLA, via inflammatory caspases, IL-18, and IFN-gamma. Eur J Immunol 45, 407–417, doi: 10.1002/eji.201444543 (2015). 25367751

[b28] Kamath A. T. . Synchronization of dendritic cell activation and antigen exposure is required for the induction of Th1/Th17 responses. J Immunol 188, 4828–4837, doi: 10.4049/jimmunol.1103183 (2012). 22504654

[b29] Li S. . Molecular signatures of antibody responses derived from a systems biology study of five human vaccines. Nat Immunol 15, 195–204, doi: 10.1038/ni.2789 (2014). 24336226 PMC3946932

[b30] Chaussabel D. & Baldwin N. Democratizing systems immunology with modular transcriptional repertoire analyses. Nat Rev Immunol 14, 271–280, doi: 10.1038/nri3642 (2014). 24662387 PMC4118927

[b31] Szabo A. . The two-component adjuvant IC31(R) boosts type i interferon production of human monocyte-derived dendritic cells via ligation of endosomal TLRs. PLoS One 8, e55264, doi: 10.1371/journal.pone.0055264 (2013). 23405128 PMC3566214

[b32] Martinez G. J. . Cutting Edge: NFAT Transcription Factors Promote the Generation of Follicular Helper T Cells in Response to Acute Viral Infection. J Immunol 196, 2015–2019, doi: 10.4049/jimmunol.1501841 (2016). 26851216 PMC4761453

[b33] Vaeth M. . Follicular regulatory T cells control humoral autoimmunity via NFAT2-regulated CXCR5 expression. J Exp Med 211, 545–561, doi: 10.1084/jem.20130604 (2014). 24590764 PMC3949566

[b34] Vogelzang A. . A fundamental role for interleukin-21 in the generation of T follicular helper cells. Immunity 29, 127–137, doi: 10.1016/j.immuni.2008.06.001 (2008). 18602282

[b35] Cantisani R. . Vaccine adjuvant MF59 promotes retention of unprocessed antigen in lymph node macrophage compartments and follicular dendritic cells. J Immunol 194, 1717–1725, doi: 10.4049/jimmunol.1400623 (2015). 25589069

[b36] Calabro S. . Vaccine adjuvants alum and MF59 induce rapid recruitment of neutrophils and monocytes that participate in antigen transport to draining lymph nodes. Vaccine 29, 1812–1823, doi: 10.1016/j.vaccine.2010.12.090 (2011). 21215831

[b37] Mastelic Gavillet B. . MF59 Mediates Its B Cell Adjuvanticity by Promoting T Follicular Helper Cells and Thus Germinal Center Responses in Adult and Early Life. J Immunol 194, 4836–4845, doi: 10.4049/jimmunol.1402071 (2015). 25870238

[b38] van Dissel J. T. . A novel liposomal adjuvant system, CAF01, promotes long-lived Mycobacterium tuberculosis-specific T-cell responses in human. Vaccine 32, 7098–7107, doi: 10.1016/j.vaccine.2014.10.036 (2014). 25454872

[b39] van Dissel J. T. . Ag85B-ESAT-6 adjuvanted with IC31 promotes strong and long-lived Mycobacterium tuberculosis specific T cell responses in naive human volunteers. Vaccine 28, 3571–3581, doi: 10.1016/j.vaccine.2010.02.094 (2010). 20226890

[b40] Lindenstrom T. . Vaccine-induced th17 cells are maintained long-term postvaccination as a distinct and phenotypically stable memory subset. Infect Immun 80, 3533–3544, doi: 10.1128/IAI.00550-12 (2012). 22851756 PMC3457559

[b41] Schraml B. U. . The AP-1 transcription factor Batf controls T(H)17 differentiation. Nature 460, 405–409, doi: 10.1038/nature08114 (2009). 19578362 PMC2716014

[b42] Qin H. . TGF-beta promotes Th17 cell development through inhibition of SOCS3. J Immunol 183, 97–105, doi: 10.4049/jimmunol.0801986 (2009). 19535626 PMC2851540

[b43] Brunner C. . BOB.1/OBF.1 controls the balance of TH1 and TH2 immune responses. EMBO J 26, 3191–3202, doi: 10.1038/sj.emboj.7601742 (2007). 17568779 PMC1914090

[b44] Caproni E. . MF59 and Pam3CSK4 boost adaptive responses to influenza subunit vaccine through an IFN type I-independent mechanism of action. J Immunol 188, 3088–3098, doi: 10.4049/jimmunol.1101764 (2012). 22351935

[b45] Mosca F. . Molecular and cellular signatures of human vaccine adjuvants. Proc Natl Acad Sci USA 105, 10501–10506, doi: 10.1073/pnas.0804699105 (2008). 18650390 PMC2483233

[b46] Morel S. . Adjuvant System AS03 containing alpha-tocopherol modulates innate immune response and leads to improved adaptive immunity. Vaccine 29, 2461–2473, doi: 10.1016/j.vaccine.2011.01.011 (2011). 21256188

[b47] Reed S. G., Orr M. T. & Fox C. B. Key roles of adjuvants in modern vaccines. Nat Med 19, 1597–1608, doi: 10.1038/nm.3409 (2013). 24309663

[b48] Kool M. . Alum adjuvant boosts adaptive immunity by inducing uric acid and activating inflammatory dendritic cells. J Exp Med 205, 869–882, doi: 10.1084/jem.20071087 (2008). 18362170 PMC2292225

[b49] Varemo L., Nielsen J. & Nookaew I. Enriching the gene set analysis of genome-wide data by incorporating directionality of gene expression and combining statistical hypotheses and methods. Nucleic acids research 41, 4378–4391, doi: 10.1093/nar/gkt111 (2013). 23444143 PMC3632109

[b50] Zhang B. & Horvath S. A general framework for weighted gene co-expression network analysis. Stat Appl Genet Mol Biol 4, Article17, doi: 10.2202/1544-6115.1128 (2005). 16646834

[b51] Langfelder P. & Horvath S. WGCNA: an R package for weighted correlation network analysis. BMC Bioinformatics 9, 559, doi: 10.1186/1471-2105-9-559 (2008). 19114008 PMC2631488

[b52] Shannon P. . Cytoscape: a software environment for integrated models of biomolecular interaction networks. Genome research 13, 2498–2504, doi: 10.1101/gr.1239303 (2003). 14597658 PMC403769

