# Supplementary material for: Comparative Systems Analyses Reveal Molecular Signatures of Clinically tested Vaccine Adjuvants
Source: Sci Rep. 2016 Dec 13;6:39097. doi: 10.1038/srep39097 (PMC5153655; doi:10.1038/srep39097)
Supplement: Supplementary Figure 1 [file srep39097-s3.doc]

**Comparative Systems Analyses Reveal Molecular Signatures of Clinically tested Vaccine Adjuvants**

**Thorunn A. Olafsdottir^1^, Madelene Lindqvist^1^, Intawat Nookaew^2,3^, Peter Andersen^4^, Jeroen Maertzdorf^5^, Josefine Persson^1^, Dennis Christensen^4^, Yuan Zhang^1^, Jenna Anderson^1^, Sakda Khoomrung^2^, Partho Sen^2^, Else Marie Agger^4^, Rhea Coler^6^, Darrick Carter^6^, Andreas Meinke^7^, Rino Rappuoli^8^**, **Stefan H. E. Kaufmann^5^, Steven G. Reed^6^, and Ali M. Harandi^1^***

^1^ Department of Microbiology and Immunology, Institute of Biomedicine, Sahlgrenska Academy, University of Gothenburg, Gothenburg, Sweden.

^2^ Department of Biology and Biological Engineering, Chalmers, University of Technology, Gothenburg, Sweden.

^3^ Department of Biomedical Informatics, College of Medicine, University of Arkansas for Medical Sciences, Little Rock, Arkansas, USA.

^4^ Department of Infectious Disease Immunology, Statens Serum Institut, Copenhagen, Denmark.

^5^ Department of Immunology, Max Planck Institute for Infection Biology, Berlin, Germany.

^6^ Infectious Disease Research Institute, Seattle, Washington, USA.

^7^ Valneva Austria GmbH, Campus Vienna Biocenter, Vienna, Austria

^8^ GSK Vaccines, Siena, Italy.

*Corresponding author: [ali.harandi@microbio.gu.se](mailto:ali.harandi@microbio.gu.se)


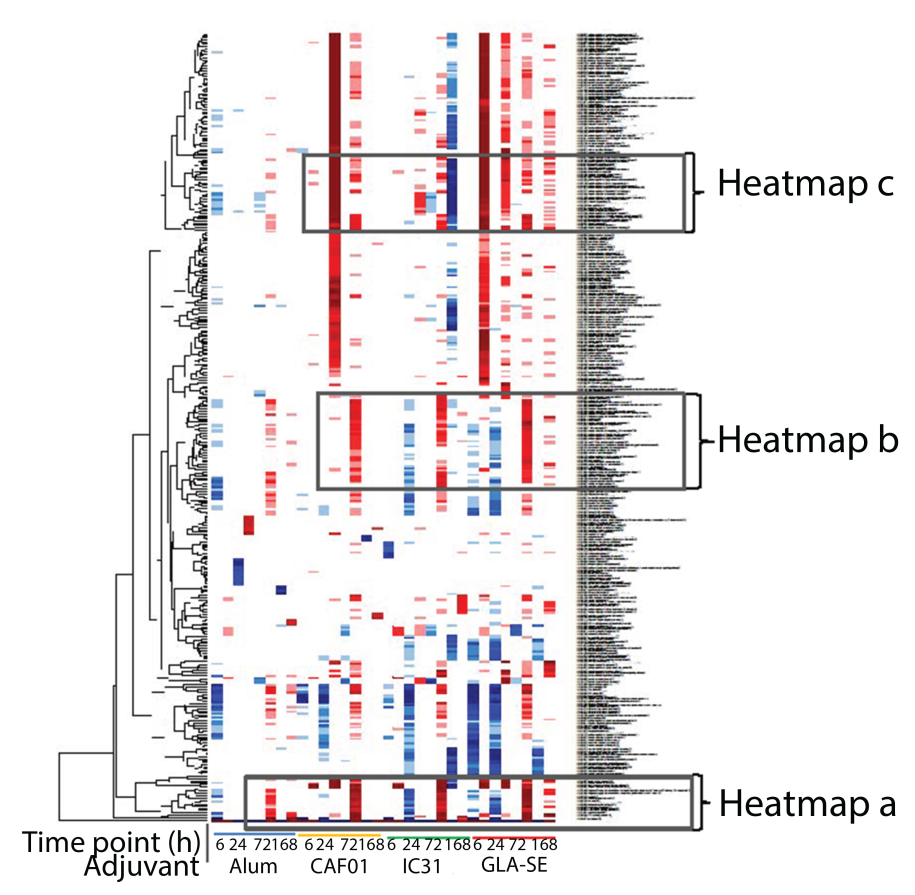


**Supplementary Figure S1: Analysis of gene ontology terms revealed shared and unique molecular signatures of vaccine adjuvants.** Heatmaps a, b and c refer to the heatmaps shown in greater detail in main figure 2.
